# Ferroptosis sensitization in glioma: exploring the regulatory mechanism of SOAT1 and its therapeutic implications

**DOI:** 10.1038/s41419-023-06282-1

**Published:** 2023-11-18

**Authors:** Shicheng Sun, Guoliang Qi, Hao Chen, Dong He, Dengzhen Ma, Yifan Bie, Linzong Xu, Bin Feng, Qi Pang, Hua Guo, Rui Zhang

**Affiliations:** 1 Department of Neurosurgery, Shandong Provincial Hospital Affiliated to Shandong First Medical University, Jinan, 250021 Shandong China; 2 Department of Neurosurgery, Shandong Provincial Hospital, Shandong University, Jinan, 250021 Shandong China; 3 Department of Radiology, The Second Hospital, Shandong University, Jinan, China; 4 Tumor Research and Therapy Center, Lanzhou University Second Hospital, Lanzhou, 730030 China; 5 Tumor Research and Therapy Center, Shandong Provincial Hospital, Shandong University, Jinan, 250021 Shandong China

**Keywords:** Genetics research, Cancer metabolism, Targeted therapies, CNS cancer, Cancer metabolism

## Abstract

Glioma, the most common primary malignant tumor of the central nervous system, lacks effective targeted therapies. This study investigates the role of SOAT1, a key gene involved in cholesterol esterification, in glioma prognosis and its association with ferroptosis. Although the impact of SOAT1 on glioma prognosis has been recognized, its precise mechanism remains unclear. In this study, we demonstrate that inhibiting SOAT1 increases the sensitivity of glioma cells to ferroptosis, both in vitro and in vivo. Mechanistically, SOAT1 positively modulates the expression of SLC40A1, an iron transporter, resulting in enhanced intracellular iron outflow, reduced intracellular iron levels, and subsequent disruption of ferroptosis. Importantly, we find that SOAT1 regulates ferroptosis independently of SREBPs, which are known to be involved in ferroptosis regulation. Furthermore, we identify the involvement of the PI3K-AKT-mTOR signaling pathway in mediating the regulatory effects of SOAT1 on SLC40A1 expression and ferroptosis sensitivity. These findings highlight the contribution of intracellular signaling cascades in the modulation of ferroptosis by SOAT1. We show that inhibiting SOAT1 enhances the efficacy of radiotherapy in gliomas, both in vitro and in vivo, by promoting sensitivity to ferroptosis. This suggests that targeting SOAT1 could potentially improve therapeutic outcomes for glioma patients. In summary, this study uncovers the pivotal role of SOAT1 as a link between cholesterol esterification and ferroptosis in glioma. Our findings underscore the potential of SOAT1 as a promising clinical therapeutic target, providing new avenues for the development of effective treatments for glioma. Further research is warranted to unravel the complete regulatory mechanisms of SOAT1 and explore its clinical applications.

## Introduction

Glioma is one of the most challenging cancers to treat [1]. Despite treatment options, including surgery, radiation, and chemotherapy, the overall prognosis of patients remains poor [2]. Complex regulatory networks have hindered the success of classic targets like p53 and epidermal growth factor receptor (EGFR). Therefore, there is a pressing need for more refined therapeutic approaches and rigorous pre-clinical trials to investigate the viability of targeted therapy [3].

Ferroptosis, an oxidative form of cell death triggered by the accumulation of iron-dependent lipid peroxides, has shown promise as a potential target for glioma therapy [4, 5]. Therefore, it is crucial to investigate approaches that can effectively modulate the sensitivity of glioma cells to targeted therapies aimed at inducing ferroptosis.

The relationship between ferroptosis and cholesterol metabolism is complex [6], and several mechanisms have been reported. In humans, the mevalonate (MVA) pathway serves as the only source of 3-isopentenylpyrophosphate IPP [7]. Tumor cells exhibit abnormal activation of the MVA pathway, resulting in elevated IPP levels. This increase in IPP content inhibits ferroptosis by modulating the maturation of GPX4 and the synthesis of ubiquinone, both of which are vital regulatory elements in ferroptosis [8–11]. Additionally, the sterol regulatory element-binding proteins (SREBPs) play a role in the cholesterol metabolism-dependent regulation of ferroptosis. SREBP1, a key transcription factor, suppresses reactive oxygen species (ROS) by transcriptionally regulating the expression of the SCD1 gene, while SREBP2 affects iron homeostasis by transcriptionally regulating Transferrin [9, 12]. Moreover, the reduction of cholesterol uptake in cholesterol-addicted cancer cells impedes the turnover of oxidized lipids, leading to the initiation of ferroptosis [13].

Increased cholesterol biosynthesis is a hallmark metabolic feature of glioma, leading to the accumulation of elevated cholesterol levels that can be esterified by sterol O-acyltransferase (SOAT) and stored as cholesteryl esters (CE) in lipid droplets [8]. In glioma, SOAT1 has been found to correlate with poor prognosis, advanced malignancy, and specific clinicopathological characteristics [14–16]. Recent studies have identified elevated expression of SOAT1 and increased cholesteryl ester content in various cancer types, including glioma, pancreatic cancer, prostate cancer, lung cancer, and adrenocortical carcinoma [17–22]. SOAT1-mediated cholesterol esterification reduces intracellular cholesterol levels, thereby relieving the inhibition of sterol regulatory element-binding proteins (SREBPs) and establishing SOAT1 as a crucial driver of cholesterol metabolism [6]. Lipid droplets were previously thought to be inert organelles, however glioblastoma has demonstrated higher SOAT1 expression compared to less malignant astrocytomas, and an inverse correlation has been observed between the number of cytoplasmic lipid droplets in gliomas and prognosis [23]. Targeting SOAT1 has been suggested as a potential therapeutic approach for GBM through the inhibition of SREBP1-mediated lipogenesis [24]. However, the relationship between SOAT1-mediated cholesterol esterification and ferroptosis remains unclear.

In our study, we investigated the impact of SOAT1 on the sensitivity of glioma cells to ferroptosis both in vivo and in vitro. We also investigated its influence on the response of glioma cells to radiotherapy, specifically through the mechanism of ferroptosis. Through our investigations, we explored the underlying mechanism and discovered that this effect was not attributable to the modulation of intracellular cholesterol balance or the activation of SREBP-mediated transcriptional regulation. Instead, we found a correlation between this effect and the PI3K-AKT-mTOR pathway. Our findings suggest that interfering with SOAT1 activity has the potential to disrupt tumor proliferation and enhance the therapeutic sensitivity induced by ferroptosis.

## Result

### SOAT1 predicts a poor prognosis, and its inhibitor avasimibe sensitizes glioma to Erastin in vivo

**Fig. 1:**
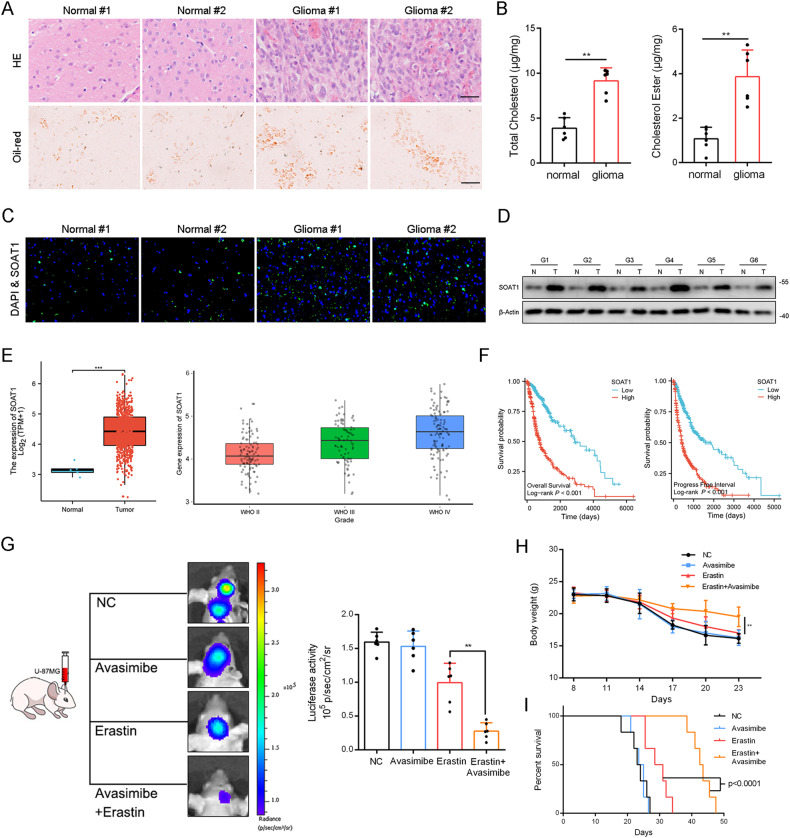
SOAT1 predicts a poor prognosis, and its inhibitor avasimibe sensitizes glioma to Erastin in vivo. **A** Human glioma tissue was stained with HE (above), oil red (below). Scale bar = 50 μm. **B** Total cholesterol and cholesterol ester content in glioma tissues were measured. **C** SOAT1 protein expression and location in human glioma tissues was marked by immunofluorescence. Scale bar = 50 μm. **D** Western blot analysis of SOAT1 protein in normal brains and paired glioma tissues. **E** Analysis of SOAT1 expression in normal brains and glioma tissues based on TCGA database data, and analysis of SOAT1 expression in glioma tissues of different grades in CGGA database. **F** The overall survival and progress free survival curve of SOAT1 expression between high and low grade gliomas was analyzed based on TCGA database data. **G**–**I** The luciferase activity of intracranial tumor, body weight and survival time of mice showed that avasimibe enhanced the effectiveness of erastin. Data significance is denoted as follows: *p < 0.05; **p < 0.01.

HE and Oil Red O staining experiments revealed that the number of cells in glioma tissue was significantly higher than that in normal tissue, and the content of lipid droplets in tissue cells was significantly increased (Fig. 1A). Total and free cholesterol were detected in the tissue cells, and the total and free cholesterol contents in glioma tissue cells were significantly higher than those in normal tissue cells (Fig. 1B). SOAT1 esterifies cholesterol into a cholesteryl ester and promotes the formation of lipid droplets [6]; therefore, we studied the expression of SOAT1 in normal brain and glioma specimens. Immunofluorescence staining was used to observe the expression changes and localization of SOAT1 in normal tissues and glioma tissues (Fig. 1C), and further western blot experiments showed that the expression of SOAT1 in glioma tissue cells was significantly increased compared with paired normal tissues (Fig. 1D).

Furthermore, by comparing the mRNA levels of SOAT1 in glioma tissues and corresponding normal tissues in a comprehensive dataset (TCGA-GBM plus TCGA-LGG), the mRNA level of SOAT1 in glioma was significantly upregulated, and the expression of SOAT1 increased with an increase in tumor grade (Fig. 1E). K-M curve analysis showed that high SOAT1 expression was associated with poor prognosis, and the survival rate of the group with high SOAT1 expression was significantly lower than that of the group with low expression (Fig. 1F).

We implanted U-87MG cells transfected with luciferase into immunocompromised mice to establish an orthotopic intracranial model. The mice were treated with the SOAT1 inhibitor avasimibe, the ferroptosis inducer erastin (20 mg/kg, IP, daily) [25] and erastin plus avasimibe (10 mg/kg, IP, daily), and luciferase activity was measured. The luciferase activity of mice treated with avasimibe was not significantly different from that of the control group. The luciferase activity of mice treated with erastin was significantly lower than that of mice treated with avasimibe and control mice, whereas that of mice treated with erastin and avasimibe was significantly lower than that of the other three groups (Fig. 1G). The body weight of the mice was further measured, and it was found that the body weight of mice treated with avasimibe alone was not significantly different from that the control group. The body weight of mice treated with erastin was higher than those of mice treated with avasimibe or the control mice. The body weights of mice treated with erastin plus avasimibe were higher than those of the mice in the other three groups (Fig. 1H). The survival cycle of mice was further measured over time, and it was found that the overall survival cycle of mice treated with avasimibe alone was not significantly different from that of the control group, and that of mice treated with erastin was longer than that of mice treated with avasimibe alone and control mice. Mice treated with erastin plus avasimibe had the longest overall survival period compared to the other three groups (Fig. 1I).

### SOAT1 inhibition sensitizes glioma cell to ferroptosis inducers

**Fig. 2:**
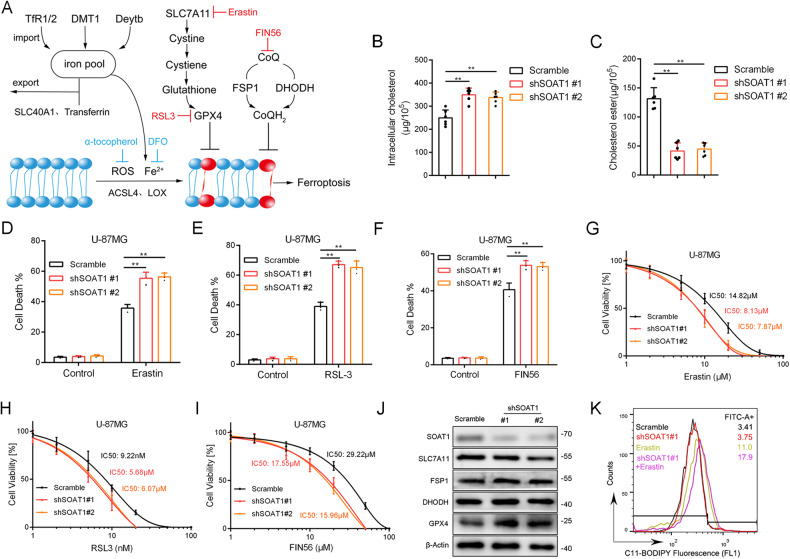
SOAT1 inhibition sensitizes glioma cell to ferroptosis inducers. **A** Schematic overview of ferroptosis pathways and iron metabolism. **B** The intracellular total cholesterol content. **C** The intracellular cholesterol ester content. **D** The SOAT1 knockout U-87MG cells showed a significant increase in cell mortality after erastin treatment. **E** After RSL-3 treatment, the SOAT1 knockout U-87MG cells significantly increased cell mortality. **F** The cell mortality rate of SOAT1 knockout U-87MG cells significantly increased when treating with FIN56. **G** Cell survival curve of U-87MG glioma cells in erastin for 24 h. **H** Cell survival curve of U-87MG glioma cells in RSL-3 for 24 h. **I** Cell survival curve of U-87MG glioma cells in FIN56 for 24 h. **J** The protein expression of key ferroptosis pathway genes SLC7A11, FSP1, DHODH, and GPX4 was measured using Western blot. The knockout of the SOAT1 gene only affects the protein expression of SOAT1. **K** Lipid peroxidation in U-87MG cells influenced by erastin and SOAT1 knockout assessed by C11-BODIPY using flow cytometry. Data significance is denoted as follows: *p < 0.05; **p < 0.01.

Ferroptosis is triggered by the accumulation of lipid peroxide [26]. We mapped key ferroptosis-related pathways, including iron ion intracellular circulation and lipid peroxidation-reduction pathways. Extracellular iron ions are transported into cells by Deytb and DMT1 and subsequently translocated into the intracellular iron pool via the action of TfR 1/2. Iron ions are then released from the iron pool, leading to intracellular iron overload. SLC40A1, which encodes ferroprotein, is an iron transporter believed to facilitate cellular iron release [27, 28]. SLC7A11 transports extracellular cysteine into cells and performs a reduction reaction to produce cysteine. Glutathione is a cofactor required for the GPX4 detoxification of peroxides, and cysteine serves as a rate-limiting raw material for glutathione [29, 30]. Ubiquinol (CoQH_2_) can detoxify lipid peroxyl radicals and is generated from ubiquinone (CoQ) in the plasma and inner mitochondrial membrane [11, 30] (Fig. 2A).

To investigate the relationship between SOAT1 and ferroptosis in gliomas, we constructed a lentivirus-mediated RNAi targeting SOAT1 (shSOAT1, #1: 5’-TTGAACTCAAGTACCAGCCTTC-3’; #2: 5′-TAATGGTCGAATTGACATAA-3′) in glioblastoma U-87MG cells and astrocytoma U-251 cells. Intracellular cholesterol in shSOAT1 cells was significantly elevated, and the cholesterol ester content was significantly reduced (Fig. 2B, C and Supplementary Fig. 1A, B). The mortality of U-87MG of shSOAT1 was significantly increased in the presence of the ferroptosis inducers erastin, RSL-3, and FIN56 (Fig. 2D–F and Supplementary Fig. 2A–C). When grown in the indicated concentrations of these inducers, cell viability was significantly lower in the shSOAT1 group, as detected by the CCK-8 toxicity assay (Fig. 2G–I).

Next, we investigated the effects of cholesterol and cholesterol esters on glioma cells and tumor-bearing mice. It was found that tumor cells treated with cholesterol (20 ug/ml) showed decreased sensitivity to ferroptosis compared with tumor cells treated with serum-free culture medium (Supplementary Fig. 3A–C). Besides, we treated cells with cholesteryl hemisuccinate (20 ug/ml) or cholesteryl oleate (10 ug/ml) and found no significant changes in ferroptosis sensitivity (Supplementary Fig. 3D). Then, we investigated the effect of hypercholesterolemia on glioma using a classical hyperlipidemia mouse model. We found no significant differences in tumor growth and ferroptosis sensitivity between hyperlipidemia group and control (Supplementary Fig. 3E–H). In addition, the study of human epidemiological showed no relationship between obesity and the risk of glioma [31].

Western blotting which was used to explore the influence of shSOAT1 on the key ferroptosis suppressor genes observed a slight decrease in SLC7A11 expression and an increase in GPX4 expression (Fig. 2J). Oxidized C11-BODIPY is generally used as a ferroptosis marker [32]. Flow cytometry and immunolabeling with oxidized C11-BODIPY showed that shSOAT1 increased erastin-induced lipid peroxidation in U-87MG cells (Fig. 2K and Supplementary Fig. 4).

### SOAT1 regulates ferroptosis sensitivity through SLC4OA1

**Fig. 3:**
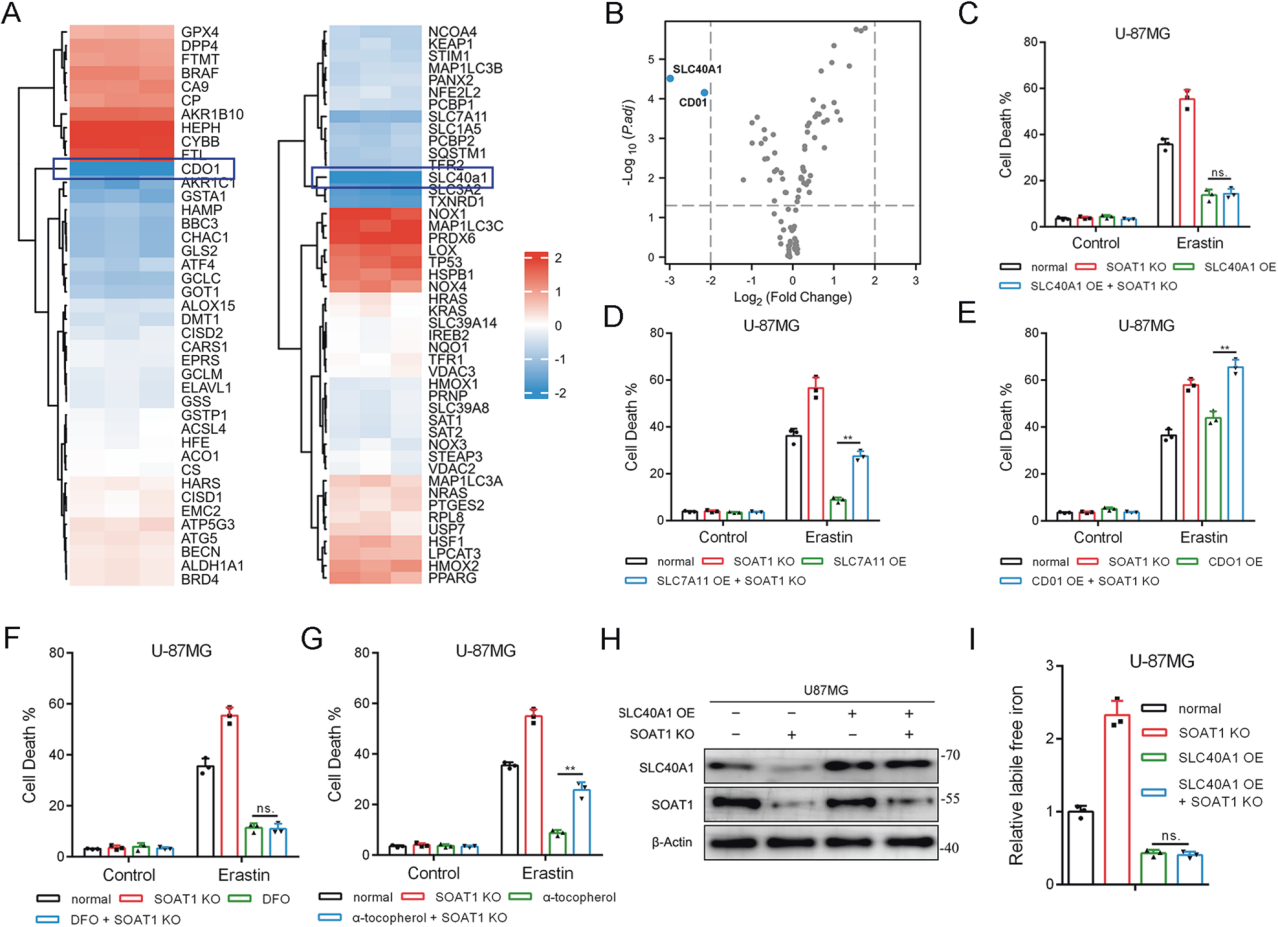
SOAT1 regulates susceptibility to ferroptosis by SLC4OA1. **A** qPCR array analyzed the downstream ferroptosis-related regulatory targets of SOAT1. Heat map showed the changes of all 87 ferroptosis-related genes. **B** Volcano map showed that SLC40A1 and CDO1 were the most related genes based on fold change and *p* value. **C**–**E** Cell death upon erastin, RSL3, and FIN56 treatment in U-87MG SOAT1 knockout cells estimated by CCK-8. **F**, **G** Cell death upon erastin when treating with DFO or tocopherol estimated by CCK-8. **H**, **I** Divided U-87MG cells into four groups to detect SLC40A1 and SOAT1 gene expressions and further measure intracellular free iron concentrations. Data significance is denoted as follows: *p < 0.05; **p < 0.01.

A qPCR array was employed to analyze the downstream regulatory targets of SOAT1, and all 87 ferroptosis-related genes with significant differences are shown in a heat map (Fig. 3A). Among all downstream ferroptosis-related genes, SLC40A1 and CDO1 were identified as the most relevant, according to folding changes and *p* values, as shown in the volcano diagram (Fig. 3B). SOAT1 knockout cells were transfected with plasmids overexpressing SLC40A1 or CDO1. The cell death rate of U-87MG cells, assessed using a CCK-8 toxicity test, was significantly higher in the SOAT1 knockout group. Notably, overexpression of SLC40A1 and SLC7A11 reversed the effect of SOAT1, whereas overexpression of CDO1 did not exhibit the same impact. Additionally, we observed that the cell death rate of the SLC40A1 overexpressing (OE) group was comparable to that of the SLC40A1 OE + SOAT1 knockout (KO) group. However, the SLC7A11 OE group differed from the SLC7A11 OE + SOAT1 KO group, indicating that SLC40A1 is the most downstream gene influencing ferroptosis, whereas SLC7A11 is not (Fig. 3C–E).

Deferoxamine (DFO), an iron scavenger, and α-tocopherol, a lipophilic antioxidant, have been reported to inhibit ferroptosis [33]. Our findings revealed that the cell death rate in the DFO group was comparable to that in the DFO + SOAT1 knockout (KO) group. Conversely, the α-tocopherol group exhibited a lower cell death rate compared to the α-tocopherol + SOAT1 KO group. These results indicate that SOAT1 regulates ferroptosis by modulating intracellular iron ions (Fig. 3F, G). After SOAT1 knockout, there was a significant increase in intracellular free iron concentration, which was significantly reduced upon SLC40A1 overexpression. Notably, the intracellular free iron levels in the SLC40A1 overexpressing (OE) group were similar to those in the SLC40A1 OE + SOAT1 KO group, suggesting that SOAT1 regulates intracellular free iron through SLC40A1 (Fig. 3H, I).

The mRNA level of SLC40A1 was significantly higher in glioma tissues than normal tissues in a comprehensive dataset (TCGA-GBM plus TCGA-LGG), and the expression of SLC40A1 increased with an increase in tumor grade (Supplementary Fig. 5A, B). K-M curve analysis showed that high SLC40A1 expression was associated with poor prognosis, and the overall survival rate and progress free survival of the group with high SLC40A1 expression was significantly lower than that of the group with low expression (Supplementary Fig. 5C). In conclusion, our findings demonstrate that SOAT1 influences the sensitivity of glioma cells to ferroptosis by modulating SLC40A1.

### SOAT1 regulated SLC40A1 in a SREBPs-independent manner

**Fig. 4:**
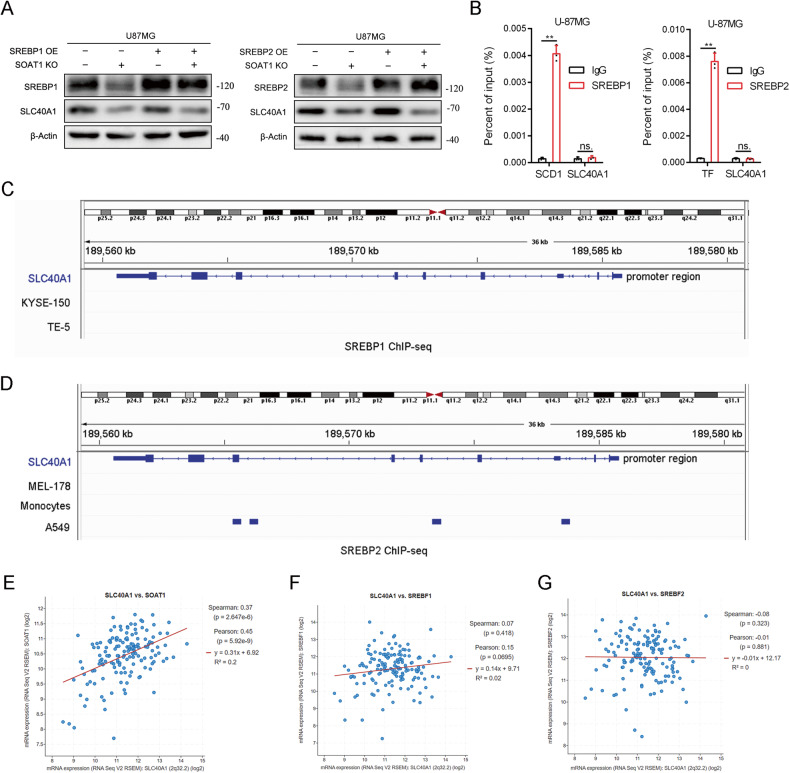
SOAT1 regulates SLC40A1 in a SREBPs-independent manner. **A** The effect of SOAT1, SREBP1, and SREBP2 on the protein expression of SLC40A1 were measured by Western blot. The positive correlation of SOAT1 affects the expression of SLC40A1, while SREBP1 and SREBP2 have no effect on the expression of SLC40A1. **B** ChIP analyses of SREBP1 and SREBP2 binding on the SLC40A1 promoter region in U-87MG cells. **C** SREBP1 ChIP-Seq showed no binding of SREBP1 on SLC40A1 promoter region in KYSE-150 and TE cells. **D** SREBP2 ChIP-Seq showed no binding of SREBP2 on SLC40A1 promoter region in MEL-178, Monocytes, and A549 cells. **E** The mRNA expression of SLC40A1 and SOAT1 had obvious positive correlation based on the TCGA datasets. **F** There was no statistically significant relationship between the mRNA expression of SLC40A1 and SREBP1. **G** There was no statistically significant relationship between the mRNA expression of SLC40A1 and SREBP2. Data significance is denoted as follows: *p < 0.05; **p < 0.01.

As a crucial enzyme in cholesterol metabolism, SOAT1 can affect the key transcription factors SREBP1 and SREBP2 in lipid metabolism by influencing intracellular cholesterol content [8]. To explore the mechanism by which SOAT1 regulates SLC40A1, we overexpressed SREP1/SREBP2 in SOAT1 knockout U-87MG cells. Western blotting confirmed that SREBP1 and SREBP2 overexpression did not reverse SLC40A1 protein levels (Fig. 4A). To explore whether SLC40A1 was downstream of SREBP1 or SREBP2, we used ChIP-qPCR to explore the direct binding relationship between SREBP1 and the SLC40A1 promoter region. SCD1 is the downstream target of the transcription factor SREBP1, and transferrin (TF) is the downstream target of SREBP2. ChIP analyses revealed strong binding of SREBP1 to SCD1 and SREBP2 on transferrin but weak binding of SREBP1 and SREBP2 to SLC40A1 in U-87MG cells (Fig. 4B). Analyses of SREBP1/SREBP2 chromatin immunoprecipitation coupled with high-throughput sequencing (ChIP-seq) datasets from GEO revealed no binding of SREBP1 on the promoter region of SLC40A1 in human esophageal squamous cell line KYSE-150 (GSM4274803) and human esophageal carcinoma cell line TE-5 (GSM4274805), while no binding of SREBP2 on the promoter region of SLC40A1 in melanoma cell line MEL-178 (GSM4774298), monocytes (GSM3702331), and lung cancer cell line A549 (GSM2423787) (Fig. 4C). Next, we found a positive correlation between SLC40A1 and SOAT1 mRNA expression in the TCGA dataset, whereas there was no statistical difference in the expression of SLC40A1 with SREBP1 and SREBP2 mRNA (Fig. 4E–G). Therefore, we concluded that SLC40A1 is not a key downstream target of SREBP1 or SREBP2.

### Regulation of SLC40A1 expression by SOAT1 is related to PI3K-AKT-mTOR signal pathway

There exists a significant association between intracellular cholesterol esters (CE) and tumor progression. Research has revealed that CE plays a role in upregulating the AKT-mTOR pathway, and inhibiting CE synthesis has been shown to suppress this signaling pathway [34–36]. Further studies have shown that PI3K-AKT-mTOR pathway affects the sensitivity of tumor cells to ferroptosis [12]. Hence, we examined whether the regulatory effect of SOAT1 on SLC40A1 was related to the AKT-mTOR signaling pathway.

**Fig. 5:**
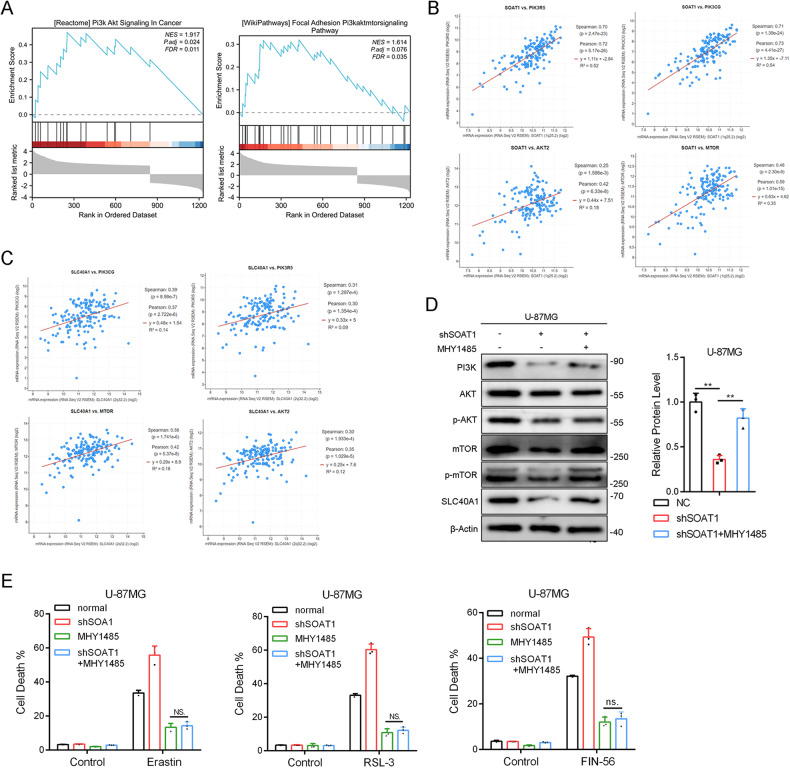
Regulation of SLC40A1 expression by SOAT1 is related to PI3K-AKT-mTOR signal pathway. **A** According to GSEA analysis, SOAT1 was related to PI3K-AKT-mTOR signal pathway. **B**, **C** SOAT1 mRNA expression was positively correlated with the mRNA expressions of PIK3R5, PIK3CG, AKT2 and mTOR, and SLC40A1 mRNA expression was also positively correlated with the mRNA expressions of PIK3R5, PIK3CG, AKT2 and mTOR. **D** Protein expression levels of PI3K, AKT, p-AKT, mTOR, p-mTOR and SLC40A1 influenced by SOAT1 knockout and mTOR agonist MHY1485. **E** Cell death upon erastin, RSL3, and FIN56 treatment with mTOR agonist MHY1485 in U-87MG SOAT1 knockout cells estimated by CCK-8. Data significance is denoted as follows: *p < 0.05; **p < 0.01.

According to GSEA analysis using TCGA datasets, SOAT1 is related to the PI3K-AKT-mTOR signaling pathway (Fig. 5A). Further information mining revealed that SOAT1 positively correlated with PIK3R5, PIK3CG, AKT2, and mTOR expression (Fig. 5B), and SLC40A1 positively correlated with PIK3R5, PIK3CG, AKT2, and mTOR mRNA expression (Fig. 5C). We explored protein changes in the PI3K/AKT/mTOR signaling pathway using western blotting. MHY-1485 is a well-known mTOR activator [35]. The results indicated that shSOAT1 silenced PI3K, phosphorylated AKT, phosphorylated mTOR, and SLC40A1 expression and that treatment with the mTOR activator MHY-1485 significantly attenuated the effect of SOAT1 knockout (Fig. 5D). Finally, MHY1485 could significantly reverse the effects of ferroptosis inducers Erastin, RSL-3 and FIN-56, and significantly reverse the enhanced sensitivity caused by SOAT1 knockout (Fig. 5E). Therefore, we concluded that the PI3K-AKT-mTOR signaling pathway is related to the effect of SOAT1 on SLC40A1 expression.

### SOAT1 sensitizes glioma to radiation by inducing ferroptosis

**Fig. 6:**
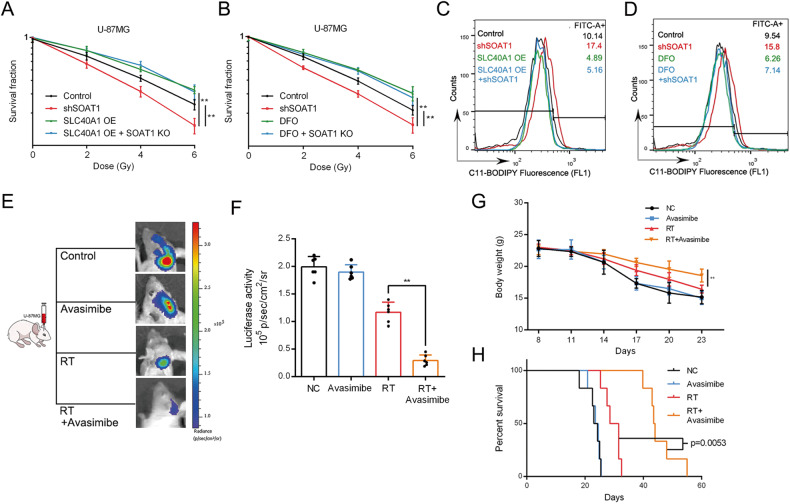
SOAT1 sensitizes glioma to radiation by inducing ferroptosis. **A** Cell survival curves at different dose of radiation. Overexpression of the SLC40A1 gene can reverse the knockout effect of the SOAT1 gene. **B** Cell survival curves at different dose of radiation. DFO can reverse the effect of SOAT1 gene knockout. **C** The effect of SOAT1 and SLC40A1 on lipid peroxidation levels at 24 h after 2 Gy irradiation in U-87MG cells using C11-BODIPY. **D** The effect of SOAT1 and DFO on lipid peroxidation levels at 24 h after 2 Gy irradiation in U-87MG cells using C11-BODIPY. **E**, **F** The luciferase activity of intracranial tumor showed that avasimibe enhanced the effectiveness of radiation. **G** The body weight of tumor-bearing mice. **H** The survival curve of tumor-bearing mice. Data significance is denoted as follows: *p < 0.05; **p < 0.01.

Ferroptosis plays a critical role in tumor radiosensitivity. SLC7A11 and GPX4, which are upregulated in tumor cells, serve to protect them from ferroptosis induced by irradiation. However, targeted inhibition of SLC7A11 or GPX4 has been shown to sensitize tumor cells to radiotherapy, leading to increased effectiveness [37–40]. In our study using the U-87MG cell line, we observed that the survival rate of the shSOAT1 group was significantly lower than that of the control group. Conversely, the survival rates of the SLC40A1 overexpression group and the SLC40A1 overexpression plus shSOAT1 group were significantly increased (Fig. 6A). Furthermore, the iron scavenger DFO displayed a similar effect to SLC40A1 overexpression, as demonstrated in Fig. 6B.

The C11-BODIPY+ cell ratios in different groups at 24 h after exposure to 2 Gy irradiation were 17.4% and 15.8% in shSOAT1, respectively; the control group was 10.14% and 9.54%, respectively, indicating a significant increase in lipid peroxidation levels after SOAT1 knockout. SLC40A1 OE and DFO were 4.89% and 6.26%, respectively, and SLC40A1 OE + shSOAT1 and DFO + shSOAT1 were 5.16% and 7.14%, respectively, indicating a decrease in lipid peroxidation levels in SLC40A1 OE, SLC40A1 OE + shSOAT1, and DFO + shSOAT1 cells (Fig. 4C, D). Therefore, silencing SOAT1 may significantly reduce the survival rate of glioma cells under RT by increasing the level of lipid peroxidation, whereas SLC40A1 OE and DFO, increases the survival rate of glioma cells by reducing the level of lipid peroxidation.

In an orthotopic intracranial model, mice were treated with avasimibe, radiotherapy (RT), or RT plus avasimibe, and luciferase activity was measured using a Xenogen IVIS Spectrum system. We found that the luciferase activity of mice treated with avasimibe was not significantly different from that of the control group. The luciferase activity of RT-treated mice was significantly lower than that of avasimibe-treated mice and control mice, whereas the activity of RT + avasimibe-treated mice was significantly lower than that of the other three groups. Luciferase activity was significantly reduced (Fig. 6E, F). The body weights of the mice were determined, and those of mice receiving RT were higher than those of mice receiving avasimibe treatment alone and control mice. Compared with the other three groups, mice treated with RT and avasimibe had the highest body weight (Fig. 6G). A survival curve of the mice revealed that mice treated with RT and avasimibe had the longest overall survival (Fig. 6H). The results showed that the gliomas were more sensitive to radiotherapy plus avasimibe treatment. In summary, high expression of SOAT1 and SLC40A1 can reduce the level of lipid peroxidation, thereby reducing the conversion of cholesterol to cholesterol esters and inhibiting ferroptosis in glioma cells.

## Discussion

The brain contains the second-highest cholesterol levels after the liver [41]. However, in the glioma microenvironment, cholesterol levels are dramatically reduced, resulting in excessive activation of MVA signaling pathways in glioma cells. These cells heavily rely on cholesterol synthesis to maintain cellular organelles and execute their functions [8]. While cholesterol levels remain relatively stable in normal cells [42], tumor cells exhibit elevated energy and biosynthetic demands associated with rapid growth [43]. Gliomas, being metabolically active tumors, exhibit elevated glycolysis, lipogenesis, and low-density lipoprotein (LDL) cholesterol uptake, which are attributed to increased lipid levels in tumor cells that promote growth [44–46]. Pre-clinical studies have indicated the impact of inhibiting cholesterol synthesis and uptake on tumor formation and growth [6], thereby revealing the intrinsic mechanisms of cholesterol metabolism in tumors.

Cholesterol homeostasis is regulated by a complex feedback mechanism, in which SREBPs play a key role. Elevated cholesterol levels can inhibit the activity of SREBPs, resulting in the inhibition of cholesterol synthesis and uptake [6, 47, 48].

The relationship between the MVA signaling pathway of cholesterol metabolism and ferroptosis is of great importance for understanding the molecular mechanisms of intracellular interactions and exploring therapeutic approaches that can interfere with both cholesterol metabolism and ferroptosis sensitivity. This provides the opportunity to regulate ferroptosis sensitivity while interfering with the cholesterol metabolic pathway, and the combined antitumor effects may have clinical application potential [49]. Cholesterol esterification is a mechanism by which the body stores and transfers cholesterol and avoids cellular toxicity caused by excess unesterified cholesterol. However, the inert conditions of CEs change dramatically if cholesterol is esterified to a polyunsaturated fatty acid or is subjected to a higher degree of enrichment. Mounting evidence has indicated that high SOAT1 expression is accompanied by high CE content in glioblastoma, pancreatic cancer, prostate cancer, and other tumors. Some studies have demonstrated that intertumoral CE accumulation is intimately linked to the proliferation and aggressive potential of breast cancer cells, and the migration of glioma cells depends on the availability of exogenous lipids and cholesterol esterification. Although the abnormal accumulation of CEs may pose a threat to male health, few studies have investigated the relationship between cholesterol esterification and ferroptosis.

We found that the key enzyme in cholesterol esterification, SOAT1, affected the sensitivity of glioma cells to ferroptosis and RT in vivo and in vitro. We found that this effect was not related to the right shift of intracellular cholesterol balance or the activation of SREBP-mediated transcriptional regulation but to the PI3K-AKT-mTOR pathway and the accumulation of CE. The regulation of SOAT1 on SLC40A1 may not be achieved through the regulation of intracellular free cholesterol levels and SREBPs but may be mediated by changes in the content or components of downstream CE. We propose a therapeutic strategy that involves the simultaneous intervention of glioma cholesterol metabolism and sensitivity to ferroptosis. Future studies should prioritize investigating the regulation of cholesterol ester content and its components, as well as exploring their relationship with targeted treatments for ferroptosis in glioma.

## Methods and materials

### Oil Red O staining

Saturated Oil Red O (G1260, solarbio, China) staining solution was used to stain the fresh frozen glioma tissues according to the reagent supplier’s program. The fresh frozen tissue sections were fixed with formaldehyde-calcium for 10 min, then fully washed in distilled water, fully soaked by 60% isopropyl alcohol (80109218, Shanghai test, CN), stained with Oil Red O solution for 10 min, differentiated into clear interstitium by 60% isopropyl alcohol (80109218, Shanghai test, CN), and then the slides were covered with glycerin gelatin.

### Western blot

Tumor tissue was removed as part of routine diagnosis and therapy. All human samples were washed twice with PBS and weighed, and then the weighed tissue was frozen with liquid nitrogen and ground until there was no tissue mass. The tissue was lysed in pre-cooling RIPA lysis buffer (P0013C, Beyotime Biotechnology, China). Tumor tissue was homogenized using an electric homogenizer and incubated on ice for 20 min. Supernatant proteins were collected by centrifugation (14,000r) for 15 min at 4 °C.

Cells were washed three times with PBS, followed by RIPA buffers containing phosphatase and protease inhibitors (P1050, Beyotime Biotechnology, Shanghai, China). An equal amount of protein samples from each group and a standard molecular weight marker were loaded on a 10% SDS-PAGE gel, followed by transferred to polyvinylidene difluoride (PVDF) membranes and blocked with 5% skimmed milk for 2 h. PVDF were probed with primary antibodies subsequent with horseradish peroxidase-conjugated secondary antibodies, then developed by an electrochemiluminescence (ECL) reagent (WBULS0100, Millipore, USA) and an enhanced chemiluminescence detection system (Amersham Imager 680). The original western blots used in this manuscript were provided in Supplementary Material.

The primary antibodies were rabbit polyclonal anti-SOAT1 (sc-20951, 1:1000, Santa Cruz, USA), rabbit polyclonal anti-SLC40A1 (ab58695, 1:1000, Abcam, USA), rabbit polyclonal anti-SLC7A11/xCT (ab60171, 1:2000, Abcam, USA), rabbit polyclonal anti-AIFM2/FSP1 (20886-1-AP, 1:1000, Proteintech, China), rabbit polyclonal anti-ACSL4 (A16848, 1:2000, ABclonal, China), rabbit polyclonal anti-GPX4 (14432-1-AP, 1:500, Proteintech, China), rabbit polyclonal anti-DHODH (14877-1-AP, 1:1000, Proteintech, China), β-actin (66009-1-Ig, 1:5000 Proteintech, China), rabbit monoclonal anti-PI3K-gamma (ab32089, 1:1000, Abcam, USA, 1/1000), rabbit monoclonal anti-AKT (4691, 1:1000, CST, USA), and rabbit monoclonal anti-p-AKT Ser473 (4060, 1:1000, CST, USA), abbit polyclonal anti-mTOR (2983, 1:1000, CST, USA).

### Flow cytometry

C11-Bodipy (D3861, 5 uM, Thermo, USA) dye to analyze lipid peroxidative level by flow cytometry. For measurements using a fluorescence microscope, obtain images using ImageXpress Micro Confocal (Molecular Devices, USA). For measurements using flow cytometry, cells were harvested in a 15 ml tube and resuspended in 500 μl PBS. Analyze the results using FACS Calibur or EPICS XL flow cytometry (BD Biosciences).

### RNA extraction and quantitative real-time PCR arrays

Total RNA was extracted from cells by a total RNA extraction kit (RC112-01, Vazyme Biotech, China). The concentration and purity of RNA was measured by absorbance at 260 nm and the ratio of 260/280 nm in NanoDrop ND-1000 (NanoDrop, Wilmington, USA). Total RNA from each samples were reversely transcribed using an all-in-one cDNA synthesis superMix (R333-01, Vazyme Biotech, China). Quantitative PCR arrays were designed to analyze a panel of ferroptosis-related genes in human glioma cell line U-87MG following the instructions of the manufacturer (Wcgene Biotechnology Corporation, China). Genes undetectable for three times were excluded. The Ct values of each gene were corrected by Ct reading of corresponding β-actin. The PCR reaction was performed using Light Cycler 480 (Roche, Switzerland).

### Orthotopic intracranial mouse model

All laboratory animal procedures are carried out in strict accordance with the “Guidelines for the Care and Use of Laboratory Animals” and approved by the Animal Care and Use Committee of Shandong Provincial Hospital Affiliated to Shandong First Medical University. Sample sizes for animal studies are based on preliminary experiments and similarly well-designed experiments, without the use of statistical methods. We used the random number method for random allocation. Male BALB/c nude mice were randomly divided into four groups, with each group consisting of six 4-week-old nude mice. In situ intracranial mouse model, each mouse was injected with 1 × 10^5^ luciferase transfected U-87MG cells. Tumor size was monitored by a heterogeneous IVIS spectroscopy system. The changes in body weight and survival of mice were monitored and recorded every day.

### Cell viability

U-87MG and U-251 cells of different groups were seeded on 96-well plates until they reached 50–60% confluency. Cells were treated with compounds including 10 μM erastin (HY-15763, MCE, USA), 10 nM RSL-3 (HY-100218A, MCE, USA), 20 μM HY-100218A (HY-14655, MCE, USA) for 24 h. Cell viability was assessed 24 h later by CCK-8. Meanwhile, cells were treated with increasing concentrations of selected drugs to draw an approximate dose-dependent toxicity curve.

### Intracellular iron determination

An iron assay kit (ab83366, Abcam, USA) was used to assess the relative iron concentration in cell lysates.

### ChIP and public transcriptional data analysis

ChIP was conducted according to the manufacturer’s procedure of ChIP-IT kit (53040, Active Motif, USA). Five million U-87MG glioma cells were collected from each group followed by immunoprecipitation of sheared DNA using 5 μl SREBP1/2 antibody. ChIP-DNA was analyzed by RT-qPCR using SYBER Green (R311-02, Vazyme Biotech, Nanjing, China). Sheared chromatin were used as Input-DNA. Primers were designed by Prime3 and validated in the Genome Browser. The results were calculated based on the CT values and analyzed as percent of input. Primer sequences used are listed in Table S1.

First from the GEO database (https://www.ncbi.nlm.nih.gov/gds/) to download esophageal squamous cancer cells (KYSE-150) (GSM4950770), human esophageal cancer cells (TE-5), MEL-178, mononuclear cells, monocytes, MEL-178 and monocytes’ CHIP-Seq data are analyzed by IGV software (IGV_2.16.0).

### Irradiation therapy

Cells were irradiated with an X-rad 320 cabinet irradiator at doses from 0 to 6 Gy. About 1000 cells/well were seeded in triplicates in six-well plates and allowed to grow from 24 h. To explore the synergistic effect of the erastin with avasimibe, cells were treated with erastin and avasimibe for 24 h. To determine the synergistic effects of avasimibe with IR, cells were pretreated with avasimibe for 24 h, then irradiated and cultured in a normal medium.

### Statistical analysis

Sample size was determined by the SEM of previous experiments. Other sample sizes were equal to or more than the general sample size recommended in previous reports. The inclusion/exclusion criteria were set in advance. If the values obtained were more than twice the SEM of the mean, we excluded the samples. Investigators were blinded during data collection and subsequent data analysis. GraphPad Prism 6.0 was used to conduct statistical analysis. All data are shown as mean ± SD from at least three independent experiments. The *t-*test was used to assess the difference between two groups. One-way ANOVA was used to assess the difference more than three groups. The indicated *p* values (*p < 0.05 and **p < 0.01) were considered statistically significant.

### Supplementary information

Supplementary figure legends

Supplementary Figure 1

Supplementary Figure 2

Supplementary Figure 3

Supplementary Figure 4

Supplementary Figure 5

Supplementary Table 1

Original Western blots

checklist

## Data Availability

The datasets used and analyzed during the current study are available from the corresponding author on reasonable request.
