# Supplementary material for: Ferroptosis sensitization in glioma: exploring the regulatory mechanism of SOAT1 and its therapeutic implications
Source: Cell Death Dis. 2023 Nov 18;14(11):754. doi: 10.1038/s41419-023-06282-1 (PMC10657441; doi:10.1038/s41419-023-06282-1)
Supplement: Supplementary file 1 — Supplementary figure legends [file 41419_2023_6282_MOESM1_ESM.docx]

**Supplementary Figure 1.** (A) Intracellular cholesterol was significantly elevated in shSOAT1 U-251 cells. (B) The cholesterol ester content was significantly reduced in shSOAT1 U-251 cells.

**Supplementary Figure 2.** (A) The SOAT1 gene knockout U-251 showed an significant increase in cell mortality after erastin treatment. (B) After RSL-3 treatment, the SOAT1 gene knockout U-251 significantly increased cell mortality. (C) After the SOAT1 gene knockout U-251 was treated with FIN56, the cell mortality rate significantly increased.

**Supplementary Figure 3.** (A-C) Cell death analysis of cholesterol-fed U-87MG cells treated with erastin (A), RSL-3 (B) and FIN56 (C). (D) Cell death analysis of cholesterol ester-fed U-87MG cells treated with erastin. (E-F) The luciferase activity of tumor-bearing mice on normal diet (ND) and high fat diet (HFD). (G) The body weight of tumor-bearing mice. (H) The survival curve of tumor-bearing mice. Data significance is denoted as follows: ∗P<0.05; ∗∗P<0.01.

**Supplementary Figure 4.** Gating strategy for lipid peroxidation analysis through flow cytometry.

**Supplementary Figure 5.** (A) Analysis of SLC40A1 expression in normal brains and glioma tissues based on TCGA database data. (B) Analysis of SLC40A1 expression in glioma tissues of different grades in CGGA database. (C) The overall survival and progress free survival curve of SLC40A1 expression between high and low grade gliomas was analyzed based on TCGA database data.
