# Supplementary figures and images for: Ferroptosis sensitization in glioma: exploring the regulatory mechanism of SOAT1 and its therapeutic implications
Source: Cell Death Dis. 2023 Nov 18;14(11):754. doi: 10.1038/s41419-023-06282-1 (PMC10657441; doi:10.1038/s41419-023-06282-1)

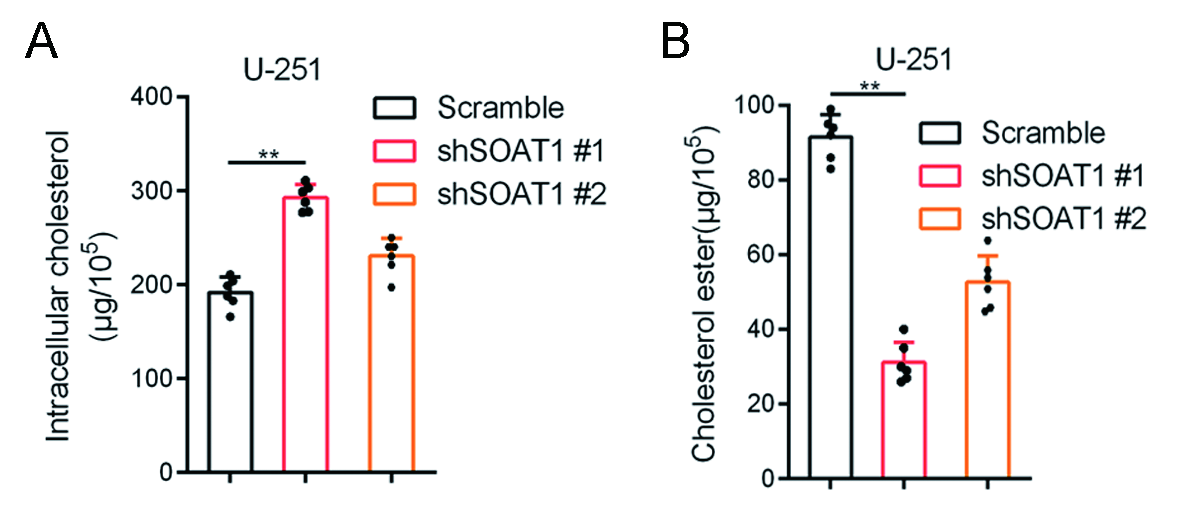

Supplement: Supplementary file 2 — Supplementary Figure 1 [file 41419_2023_6282_MOESM2_ESM.tif]

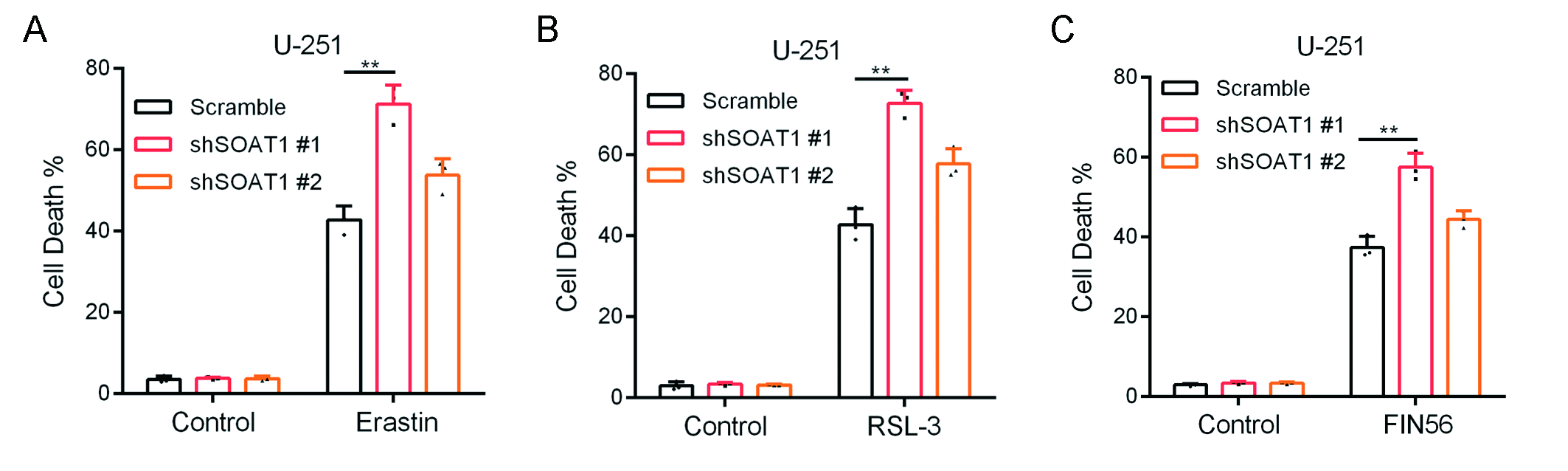

Supplement: Supplementary file 3 — Supplementary Figure 2 [file 41419_2023_6282_MOESM3_ESM.tif]

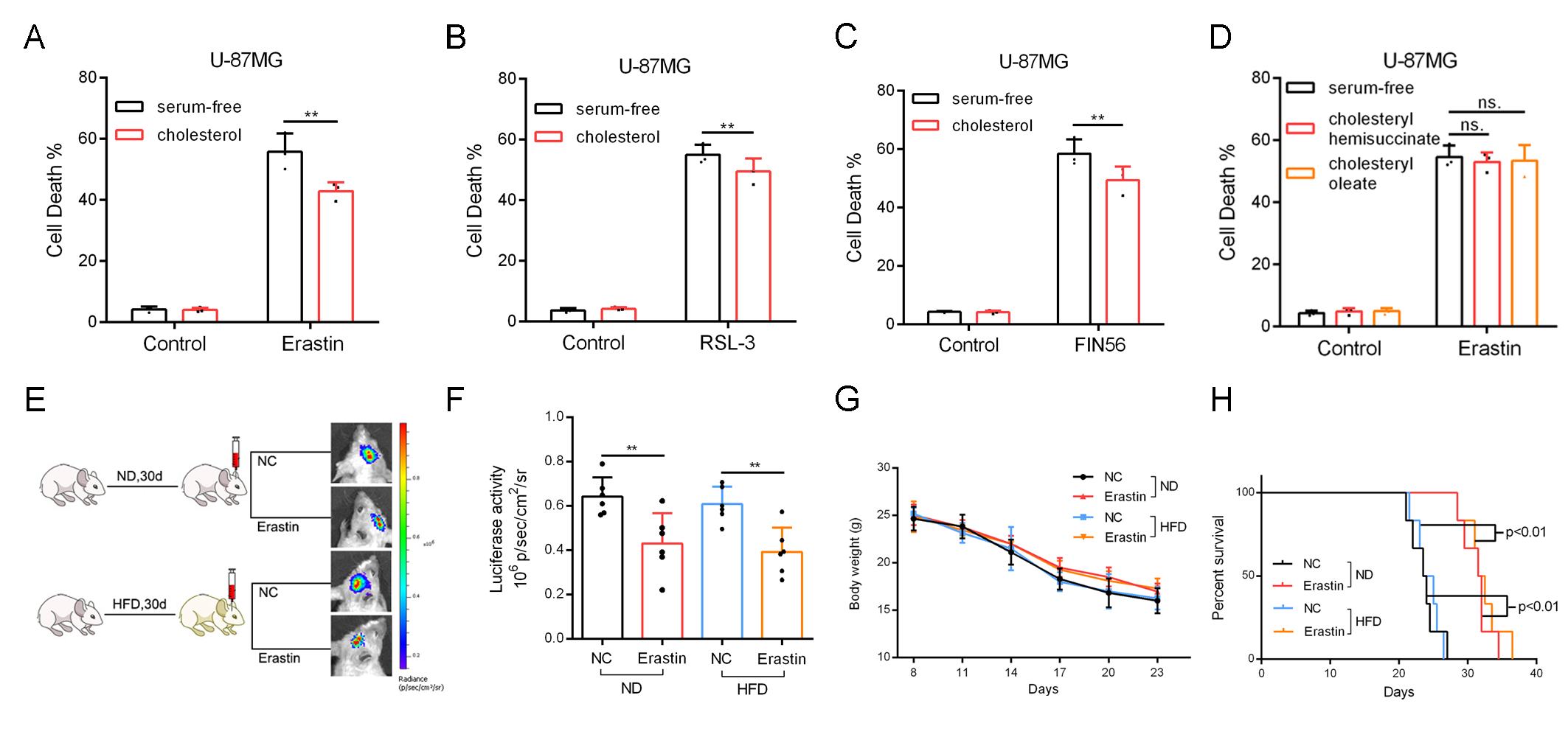

Supplement: Supplementary file 4 — Supplementary Figure 3 [file 41419_2023_6282_MOESM4_ESM.tif]

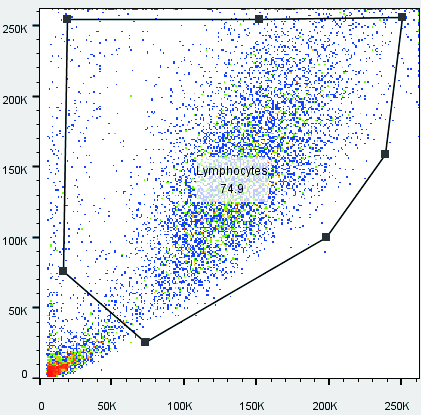

Supplement: Supplementary file 5 — Supplementary Figure 4 [file 41419_2023_6282_MOESM5_ESM.tif]

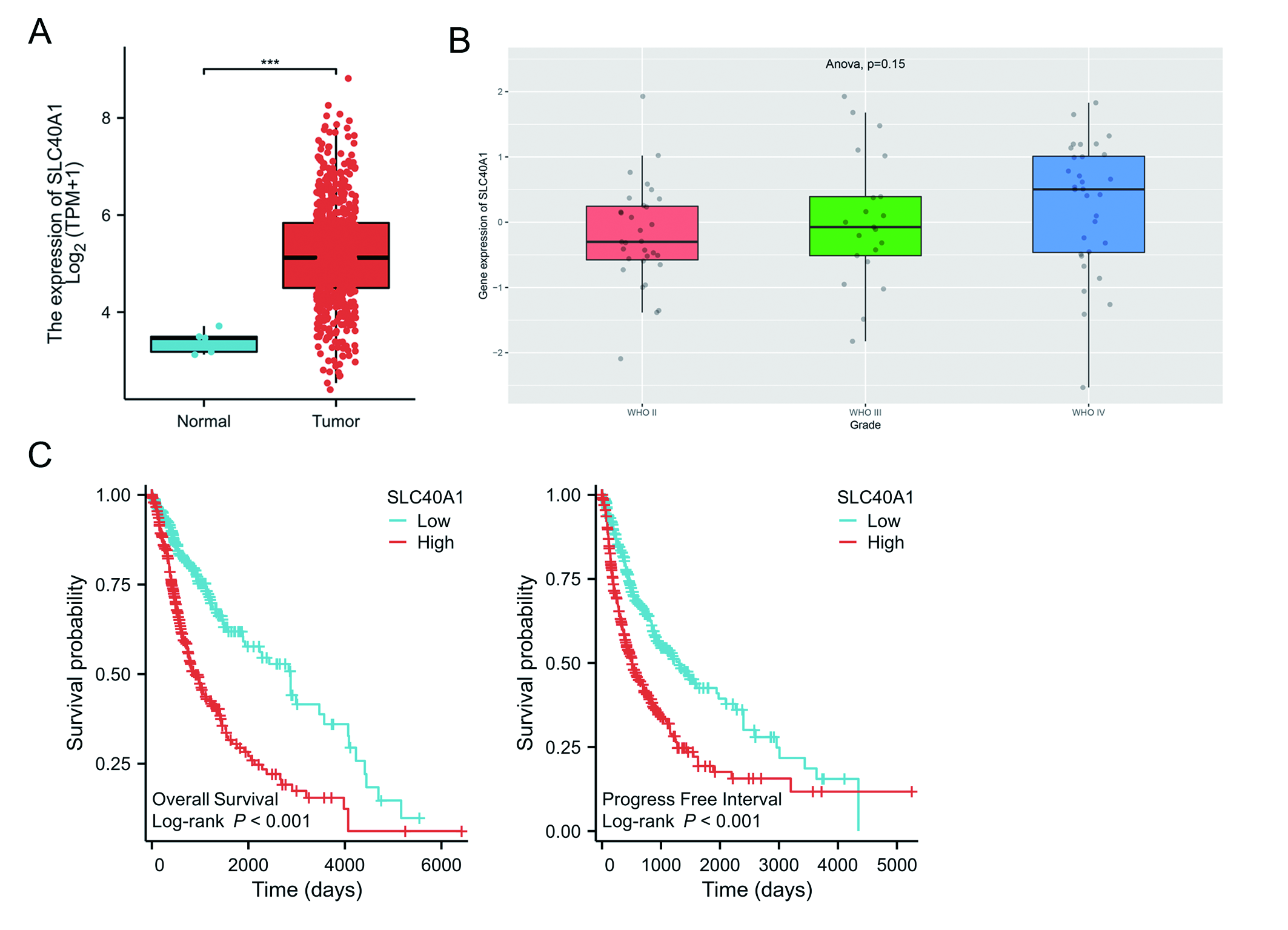

Supplement: Supplementary file 6 — Supplementary Figure 5 [file 41419_2023_6282_MOESM6_ESM.tif]

Figure 1D


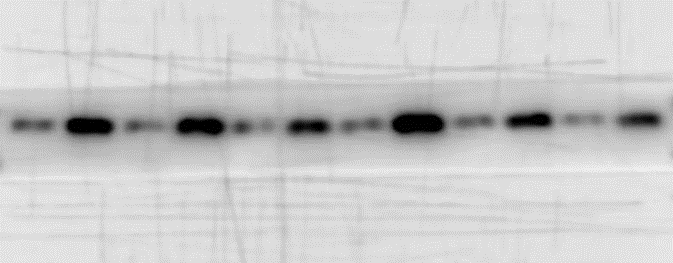


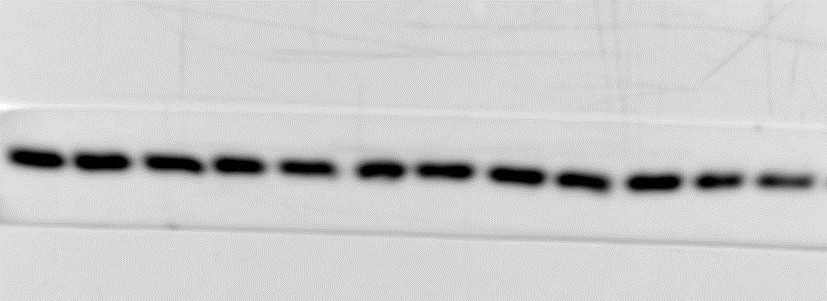


Figure 2J


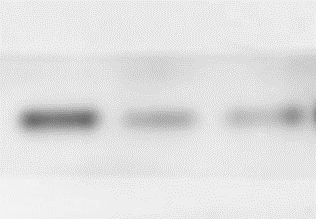


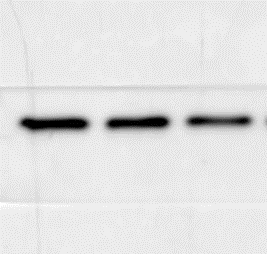


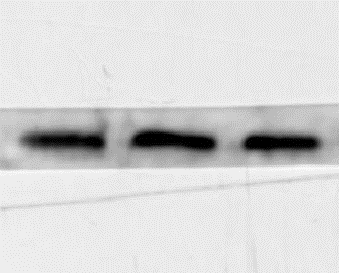


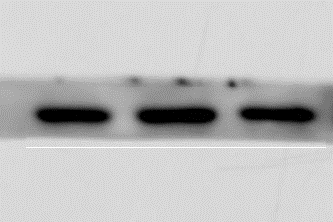


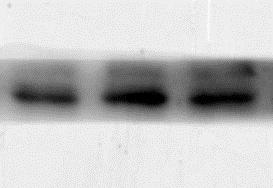


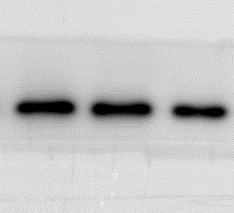


Figure 3H


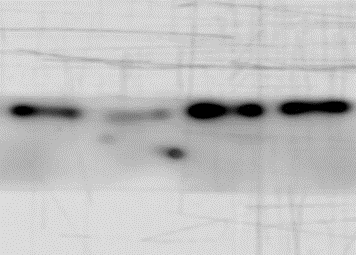


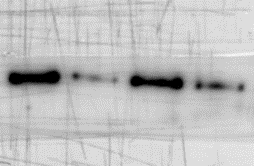


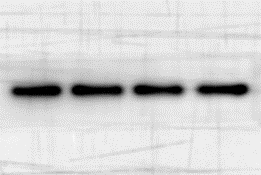


Figure 4A


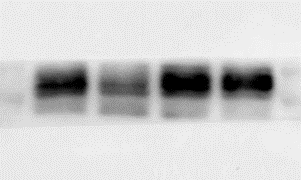


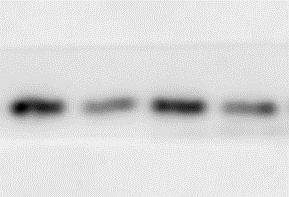


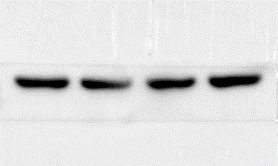


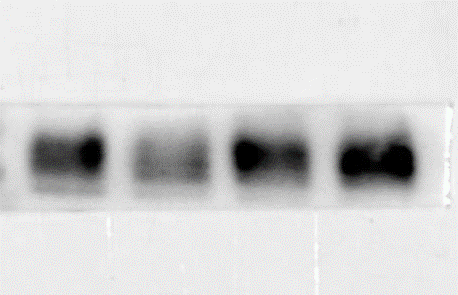


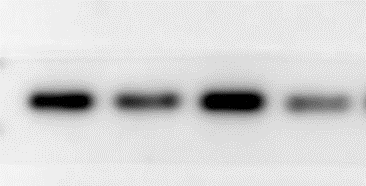


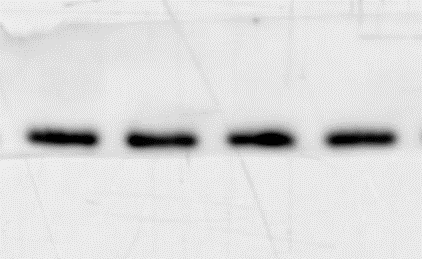


Figure 5D


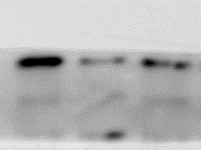


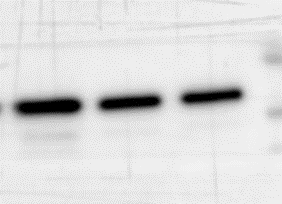


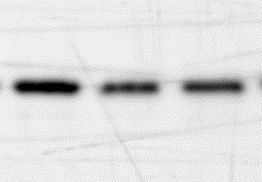


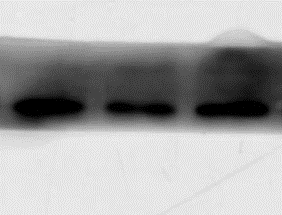


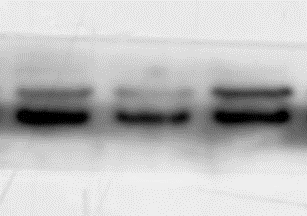


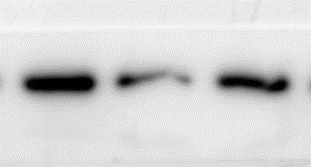


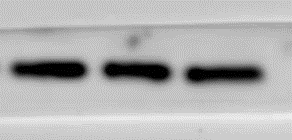

Supplement: Supplementary file 8 — Original Western blots [file 41419_2023_6282_MOESM8_ESM.docx]
