# Supplementary material for: Ferroptosis sensitization in glioma: exploring the regulatory mechanism of SOAT1 and its therapeutic implications
Source: Cell Death Dis. 2023 Nov 18;14(11):754. doi: 10.1038/s41419-023-06282-1 (PMC10657441; doi:10.1038/s41419-023-06282-1)
Supplement: Supplementary file 7 — Supplementary Table 1 [file 41419_2023_6282_MOESM7_ESM.docx]

| **Supplementary Table S1. Primers used in this study** | | |
| --- | --- | --- |
| **Genes** | **Primer sequence (5’-3’)** | |
| ***SOAT1***  ***(Human)*** | F: CCACTGGTCCAGATGAGTTTAG | R: GGGAACATGCAGAGTACCTTT |
| ***SLC7A11***  ***(Human)*** | F: CTTTGTTGCCCTCTCCTGCTTC | R: CAGAGGAGTGTGCTTGTGGACA |
| ***SLC40A1***  ***(Human)*** | F: AACAAGCACCTCAGCGAGAG | R: AACAAGCACCTCAGCGAGAG |
| ***β-actin***  ***(Human)*** | F: AGGCCAACCGCGAGAAGATGACC | R: GAAGTCCAGGGCGACGTAGCAC |
| ***SLC40A1***  ***promoter***  ***(Human)*** | F: CTATCGATAGGTACCGCTATGGTTCAC  AGCAGAGC | R: ATCGCAGATCTCGAGGGATTTAAGA  TTCCCCTTCCACAG |
